# A review of Canadian and Alaskan species of the genera *Clusiota* Casey and Atheta Thomson, subgenus Microdota Mulsant & Rey (Coleoptera, Staphylinidae, Aleocharinae)

**DOI:** 10.3897/zookeys.524.6105

**Published:** 2015-09-30

**Authors:** Jan Klimaszewski, Reginald P. Webster, Derek Sikes, Caroline Bourdon, Myriam Labrecque

**Affiliations:** 1 Natural Resources Canada, Canadian Forest Service, Laurentian Forestry Centre, 1055 du P.E.P.S., P.O. Box 10380, Stn. Sainte-Foy, Québec, Quebec, Canada G1V 4C7; 2 24 Mill Stream Drive, Charters Settlement, New Brunswick, Canada E3C 1X1; 3 University of Alaska Museum, 907 Yukon Dr., Fairbanks, Alaska, USA, 99775-6960

**Keywords:** Alaska, Canada, Coleoptera, Staphylinidae, Aleocharinae, *Clusiota*, *Microdota*, new records, adventive species

## Abstract

This paper treats 13 species of the subgenus *Microdota* Mulsant & Rey of *Atheta* Thomson and 3 species of the genus *Clusiota* Casey in Canada and Alaska. We report here 4 species new to science, and 3 new provincial records. The following species are new to science: Atheta (Microdota) curtipenis Klimaszewski & Webster, **sp. n.**, Atheta (Microdota) formicaensis Klimaszewski & Webster, **sp. n.**, Atheta (Microdota) macesi Klimaszewski & Webster, **sp. n.**, and *Clusiota grandipenis* Klimaszewski & Webster, **sp. n.** The new provincial records are: Atheta (Microdota) pseudosubtilis Klimaszewski & Langor, new to AB, and Atheta (Microdota) subtilis (Scriba), an adventive Palaearctic species new to North America, first reported in LB and NB. The two *Clusiota* Casey species are reviewed, and their distribution is revised. A female *Clusiota impressicollis* was discovered in Ontario and is illustrated here for the first time. A key to all Canadian species of the subgenus *Microdota* and genus *Clusiota* are provided. Atheta (Microdota) holmbergi Bernhauer and Atheta (Microdota) alesi Klimaszewski & Brunke are transferred here to the subgenus *Dimetrota* Mulsant & Rey.

## Introduction

Aleocharines are species rich in the boreal forest of Canada but knowledge of them, despite recent progress (Klimaszewski et al. 2015), is still fragmentary and there are many species likely to be discovered as new to science or as new records of adventive or formerly known species from the USA (Klimaszewski et al. 2015).

This paper deals with Canadian species of the subgenus *Microdota* Mulsant and Rey of the genus *Atheta* Thomson and Rey, and the genus *Clusiota* Casey occurring in Canada and Alaska. The subgenus *Microdota* contains about 215 species in the Palaearctic region (Lee and Ahn 2015). In the Nearctic region the true number of species is unknown but Ashe (2000) reported 27 species. *Microdota* species may be confused with those of *Clusiota* due to their small size, and other superficial similarities. That is why both groups are treated here. *Microdota* species may also be confused with members of the subgenus *Datomicra* Mulsant and Rey from which they may be separated by having a fully exposed pronotal hypomeron in lateral view, whereas in *Datomicra* it is only partially exposed (Seevers 1978, Ashe 2000). In Canada we recognize 13 *Microdota* species including 3 species described here as new to science, and 3 species of *Clusiota*, including one species new to science. These *Clusiota* species constitute all known Nearctic species of the genus. We provide diagnoses of new or newly recorded species, illustrations of habitus and genital structures of all *Microdota* species, and keys to their identification. We hope that this publication will lead to the proper identification of species in this difficult group and will make them available for ecological, environmental, and other studies.

## Materials and methods

About 100 adults of the genera *Microdota* and *Clusiota* from Canada and Alaska were studied, and most specimens were dissected to examine the genitalic structures that were dehydrated in absolute alcohol, mounted in Canada balsam on celluloid microslides, and pinned with the specimens from which they originated. Images of the entire body and the genital structures were taken using an image processing system (Nikon SMZ 1500 stereoscopic microscope; Nikon Digit-like Camera DXM 1200F, and Adobe Photoshop software).

Morphological terms mainly follow those used by Seevers (1978), Ashe (2000), and Klimaszewski et al. (2011). The ventral side of the median lobe of the aedeagus is considered to be the side of the bulbus containing the foramen mediale, the entrance of the ductus ejaculatorius, and the adjacent ventral side of the tubus of the median lobe with internal sac and its structures (this part is referred to as the parameral side in some recent publications); the opposite side is referred to as the dorsal part. In the species descriptions, microsculpture refers to the surface of the upper forebody (head, pronotum and elytra).

**Distribution.** Each species is cited with its currently known distribution in Canada and Alaska. The following abbreviations are used in the text for Canadian provinces and territories:

AB – Alberta, BC – British Columbia, LB – Labrador, MB – Manitoba, NB – New Brunswick, NF – Newfoundland (island), NS – Nova Scotia, NT – Northwest Territories, NU – Nunavut, ON – Ontario, PE – Prince Edward Island, QC – Quebec, SK – Saskatchewan, YT – Yukon Territory.

USA state abbreviations follow those of the US Postal Service.

Two labels were used on some specimens (RWC), one that included the locality, collection date, and collector, and one with macro and micro habitat data and collection method. Information from the two labels is separated by a // in the data presented for these specimens.

### Depository/institutional abbreviations

CNC Canadian National Collection of Insects, Arachnids and Nematodes, Agriculture and Agri-Food Canada, Ottawa, Ontario, Canada.

LFC Natural Resources Canada, Canadian Forest Service, Laurentian Forestry Centre, R. Martineau Insectarium, Quebec City, Quebec, Canada.

RWC Reginald Webster Collection, Charters Settlement, New Brunswick, Canada.

UAM University of Alaska Museum, University of Alaska, Fairbanks, Alaska, U.S.A. http://dx.doi.org/doi: 10.7299/X75D8S0H

ZMB Zoological Museum of Humboldt University, Berlin, Germany.

ZML Museum of Zoology, Lund University, Lund, Sweden.

## Checklist of Canadian *Microdota* and *Clusiota* species

New jurisdictional records are indicated in bold type.

**Genus *Atheta* Thomson, 1858**

**Subgenus *Microdota* Mulsant & Rey, 1873**

1) Atheta (Microdota) amicula (Stephens, 1832). Palaearctic; adventive in Canada: NF, NS. USA: WA.

2) **Atheta (Microdota) curtipenis** Klimaszewski & Webster, **sp. n. Canada: NB**.

3) Atheta (Microdota) festinans (Erichson, 1839). Canada: ON. USA: IN, ME, MI, PA.

4) **Atheta (Microdota) formicaensis** Klimaszewski & Webster, **sp. n. Canada: NB**.

5) **Atheta (Microdota) macesi** Klimaszewski & Webster, **sp. n. Canada: NB.**

6) Atheta (Microdota) microelytrata Klimaszewski & Godin, 2012. Canada: YT.

7) Atheta (Microdota) pennsylvanica Bernhauer, 1907. Canada: LB, NB, NF, NS, ON, QC. USA: IN, PA, RI, VA.

8) Atheta (Microdota) platonoffi Brundin, 1948. Holarctic; Canada: AB, BC, LB, NB, NF, NS, ON, YT. USA: AK.

9) Atheta (Microdota) pratensis (Mäklin, 1852). USA: AK, WA.

10) Atheta (Microdota) pseudosubtilis Klimaszewski & Langor, 2011. Canada: **AB**, LB, NB, NF, QC, YT.

11) Atheta (Microdota) riparia Klimaszewski & Godin, 2012. Canada: YT.

12) Atheta (Microdota) sculptisoma Klimaszewski & Langor, 2011. Canada: NF, QC.

13) Atheta (Microdota) subtilis (Scriba, 1866). Palaearctic, adventive in **Canada: LB, NB.**

**Species transferred to the subgenus *Dimetrota* Mulsant & Rey**

14) Atheta (Microdota) alesi (Klimaszewski & Brunke, 2012). Canada: ON.

15) Atheta (Microdota) holmbergi Bernhauer, 1907. Canada: BC. USA: AK.

**Species removed from NF and LB species list** (misidentification for *Atheta subtilis* Mulsant and Rey)

16) Atheta (Microdota) pratensis (Mäklin, 1852). Canada: YT. USA: AK.

**Subgenus *Clusiota* Casey, 1910**

17) *Clusiota antennalis* Klimaszewski and Godin, 2008. Canada: **BC**. USA: AK.

18) *Clusiota impressicollis* (Bernhauer, 1907). Canada: BC, **ON**, NB, NF.

19) ***Clusiota grandipenis*** Klimaszewski and Webster, **sp. n. Canada: NB**.

## Taxonomic review

### Genus *Atheta* Thomson

#### Subgenus Microdota

Taxon classificationAnimaliaColeopteraStaphylinidae

Mulsant & Rey

Microdota (Mouthparts illustrated by Lee and Ahn 2015)

##### Diagnosis.

The following combination of characters is distinctive for *Microdota*: small and subparallel body (Figs 1, 9, 17, 25, 33, 41, 49, 56, 61, 69, 80, 84), length 1.5−2.7 mm, antennomere I enlarged but not extremely swollen, longer than II, II longer than III, V−X slightly to strongly transverse; median region of prementum very narrow and without pseudopores; labial palpus with 3 articles; glossa split apically forming Y-shaped structure; maxillary palpus with 3 articles, last one narrowly elongate; pronotum transverse, more than 1.2 times as wide as long, midline pubescence directed in most specimens anteriorly and laterad elsewhere (Figs 1, 9, 17, 25, 33, 41, 49, 56, 61, 69, 80, 84); pronotal hypomeron fully visible laterally; elytra in some species with wavy pattern of pubescence in postero-sutural section of disc (Figs 33, 49); median lobe of aedeagus with large bulbus and triangularly shaped apex of tubus in dorsal view, internal sac of median lobe of aedeagus with well-developed complex structures (Figs 4, 5, 12, 13, 20, 21, 28, 29, 36, 37, 44, 45, 51, 52, 64, 65, 72–75, 87); spermatheca of variable shape, L- or S-shaped, capsule usually in a form of a narrow sac or club-shaped, and stem often sinuate (Figs 8, 16, 24, 32, 40, 48, 55, 59, 60, 68, 78, 79, 83); male tergite VIII in most species simple, truncate apically and without teeth, sometimes with minute crenulation and small pairs of teeth (Figs 2, 10, 18, 26, 34, 42, 50, 62, 70, 85).

**Figures 1–8.**
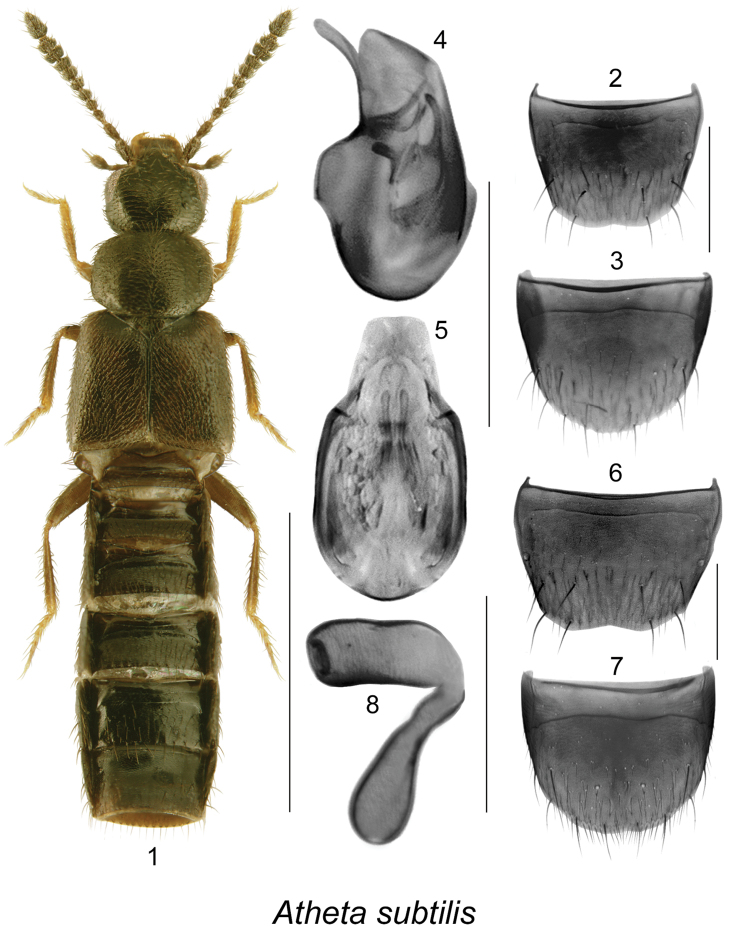
Atheta (Microdota) subtilis (Scriba): **1** habitus in dorsal view **2** male tergite VIII **3** male sternite VIII **4** median lobe of aedeagus in lateral view **5** median lobe of aedeagus in dorsal view **6** female tergite VIII **7** female sternite VIII **8** spermatheca. Scale bar for habitus = 1 mm; remaining scale bars = 0.2 mm.

**Figures 9–16.**
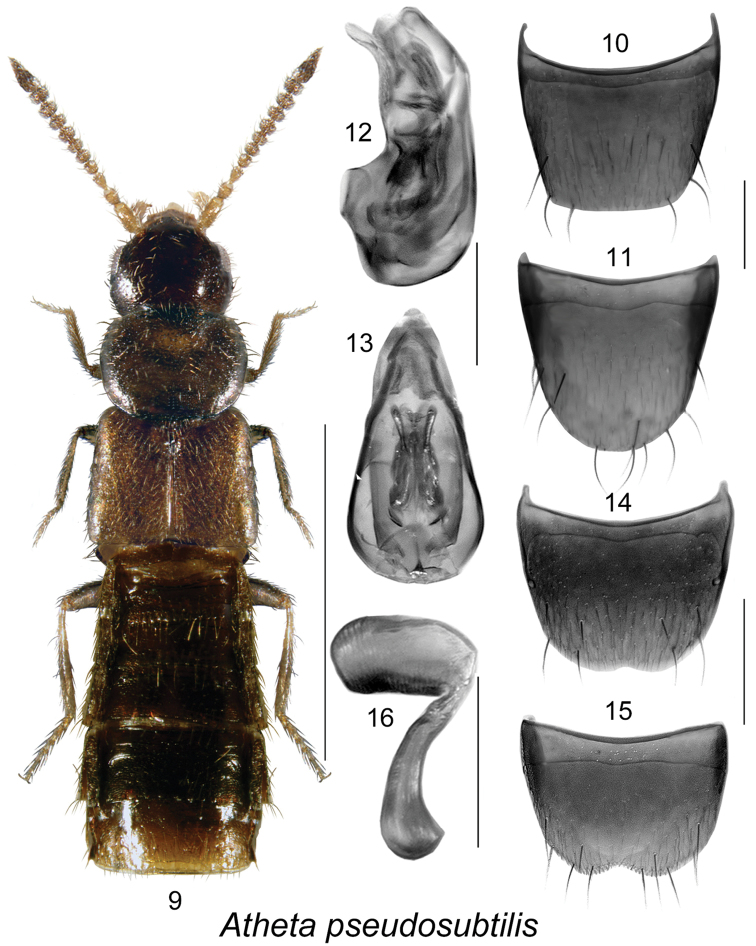
Atheta (Microdota) pseudosubtilis Klimaszewski & Langor: **9** habitus in dorsal view **10** male tergite VIII **11** male sternite VIII **12** median lobe of aedeagus in lateral view **13** median lobe of aedeagus in dorsal view **14** female tergite VIII **15** female sternite VIII **16** spermatheca. Scale bar for habitus = 1 mm; remaining scale bars = 0.2 mm.

**Figures 17–24.**
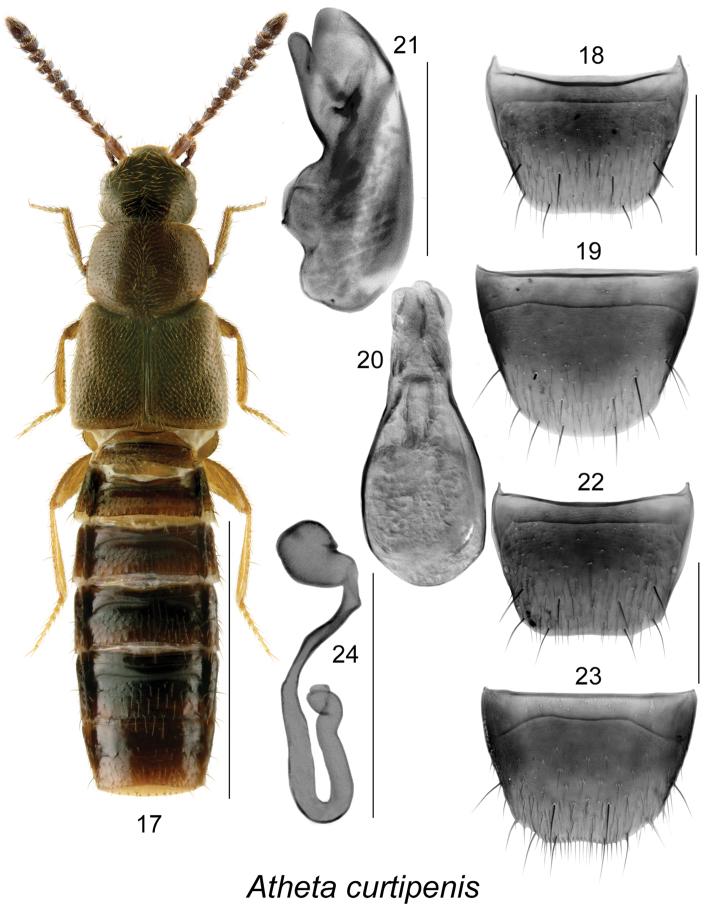
Atheta (Microdota) curtipenis Klimaszewski & Webster, sp. n.: **17** habitus in dorsal view **18** male tergite VIII **19** male sternite VIII **20** median lobe of aedeagus in dorsal view **21** median lobe of aedeagus in lateral view **22** female tergite VIII **23** female sternite VIII **24** spermatheca. Scale bar for habitus = 1 mm; remaining scale bars = 0.2 mm.

**Figures 25–32.**
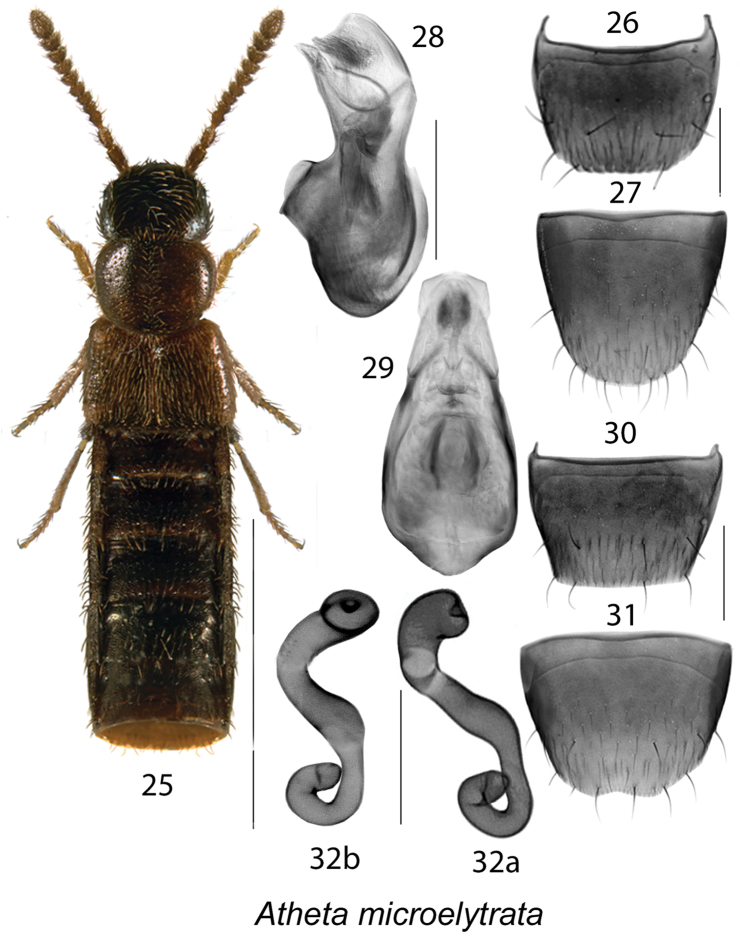
Atheta (Microdota) microelytrata Klimaszewski & Godin: **25** habitus in dorsal view **26** male tergite VIII **27** male sternite VIII **28** median lobe of aedeagus in lateral view **29** median lobe of aedeagus in dorsal view **30** female tergite VIII **31** female sternite VIII **32a, b** spermatheca. Scale bar for habitus = 1 mm; remaining scale bars = 0.2 mm.

**Figures 33–40.**
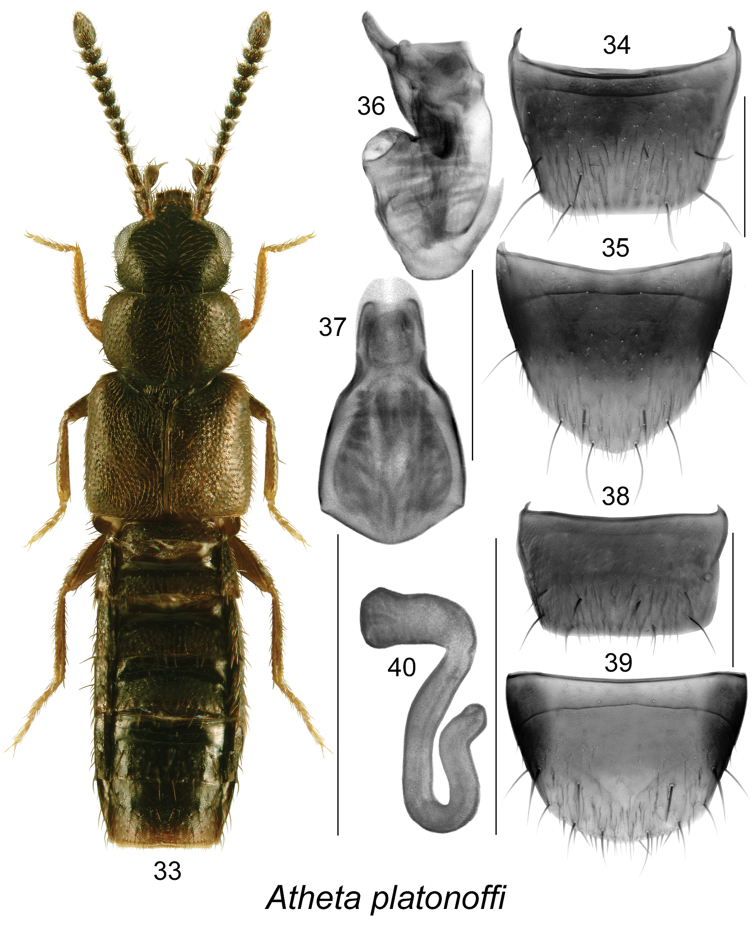
Atheta (Microdota) platonoffi Bernhauer: **33** habitus in dorsal view **34** male tergite VIII **35** male sternite VIII **36** median lobe of aedeagus in lateral view **37** median lobe of aedeagus in dorsal view **38** female tergite VIII **39** female sternite VIII **40** spermatheca. Scale bar for habitus = 1 mm; remaining scale bars = 0.2 mm.

**Figures 41–48.**
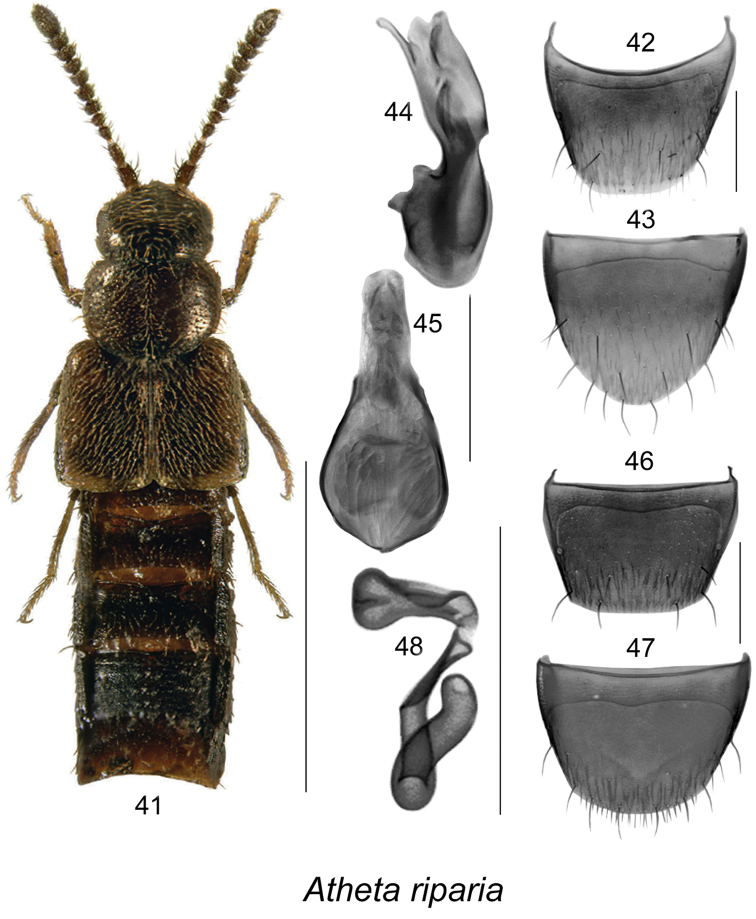
Atheta (Microdota) riparia Klimaszewski & Godin: **41** habitus in dorsal view **42** male tergite VIII **43** male sternite VIII **44** median lobe of aedeagus in lateral view **45** median lobe of aedeagus in dorsal view **46** female tergite VIII **47** female sternite VIII **48** spermatheca. Scale bar for habitus = 1 mm; remaining scale bars = 0.2 mm.

**Figures 49–55.**
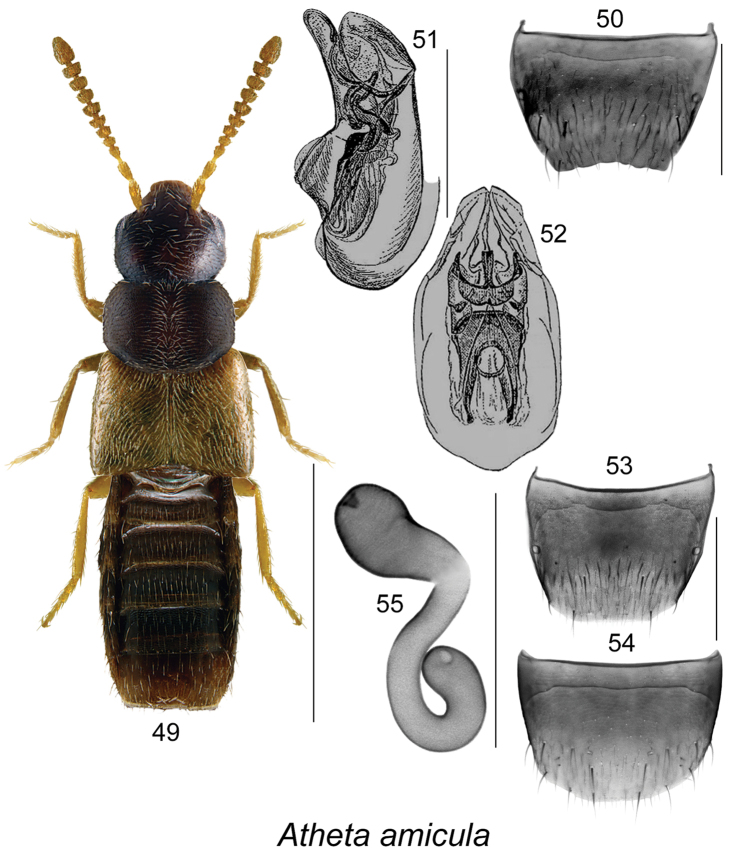
Atheta (Microdota) amicula (Stephens): **49** habitus in dorsal view **50** male tergite VIII (based on European specimen) **51** median lobe of aedeagus in lateral view (after Brundin 1948) **52** median lobe of aedeagus in dorsal view (after Brundin 1948) **53** female tergite VIII **54** female sternite VIII **55** spermatheca. Scale bar for habitus = 1 mm; remaining scale bars = 0.2 mm.

**Figures 56–60.**
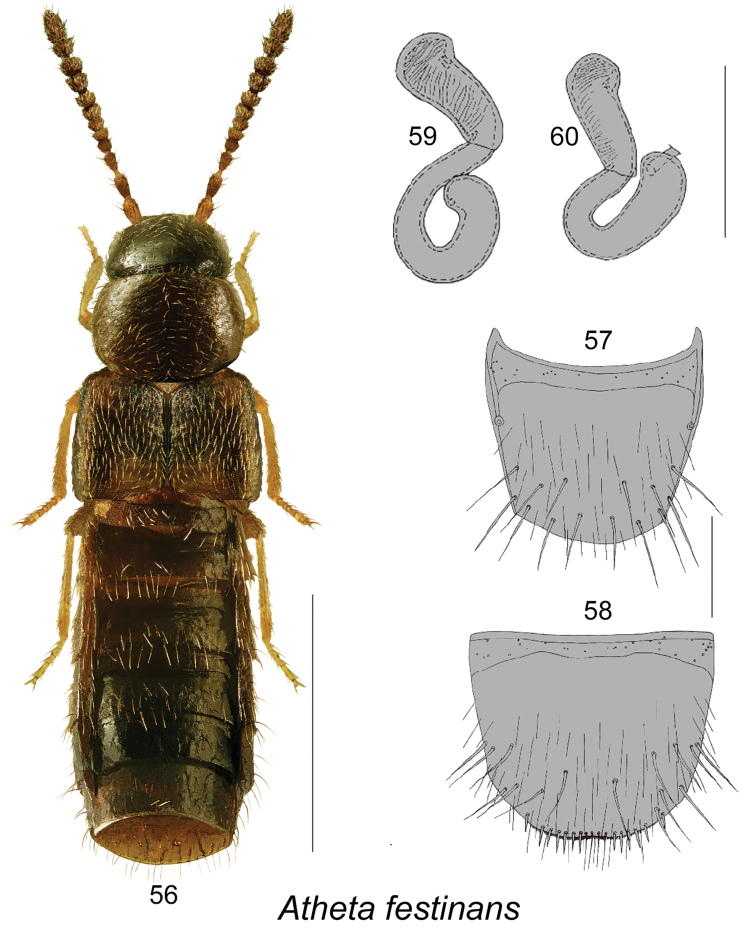
Atheta (Microdota) festinans (Erichson): **56** habitus in dorsal view **57** female tergite VIII **58** female sternite VIII **59, 60** spermatheca. Scale bar for habitus = 1 mm; remaining scale bars = 0.2 mm.

**Figures 61–68.**
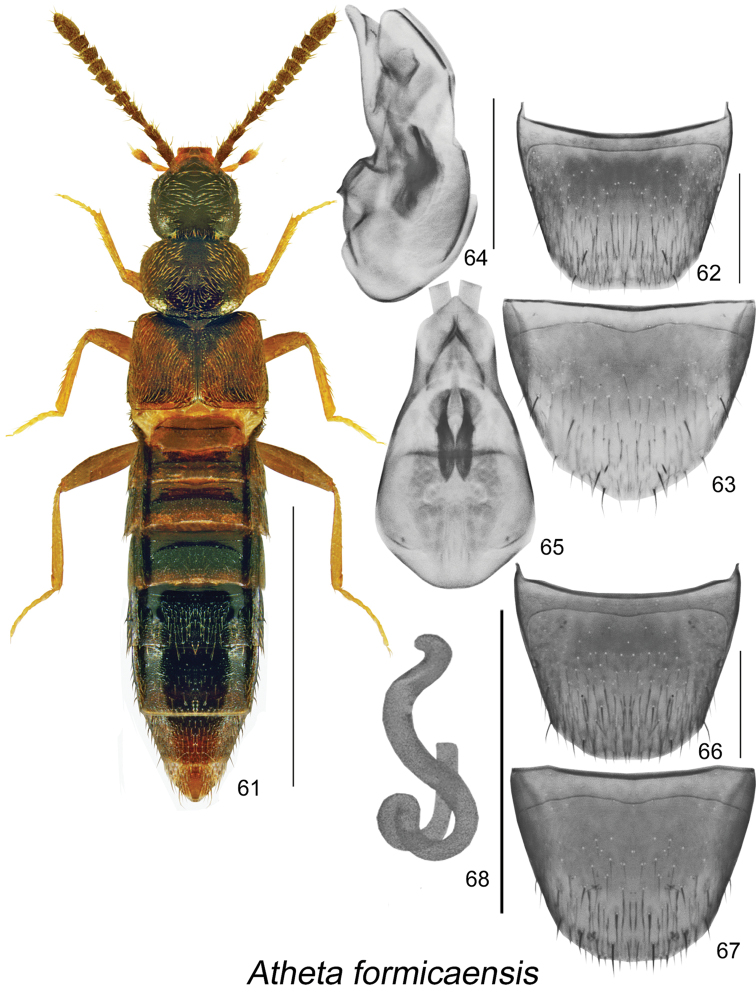
Atheta (Microdota) formicaensis Klimaszewski & Webster, sp. n.: **61** habitus in dorsal view **62** male tergite VIII **63** male sternite VIII **64** median lobe of aedeagus in lateral view **65** median lobe of aedeagus in dorsal view **66** female tergite VIII **67** female sternite VIII **68** spermatheca. Scale bar for habitus = 1 mm; remaining scale bars = 0.2 mm.

**Figures 69–79.**
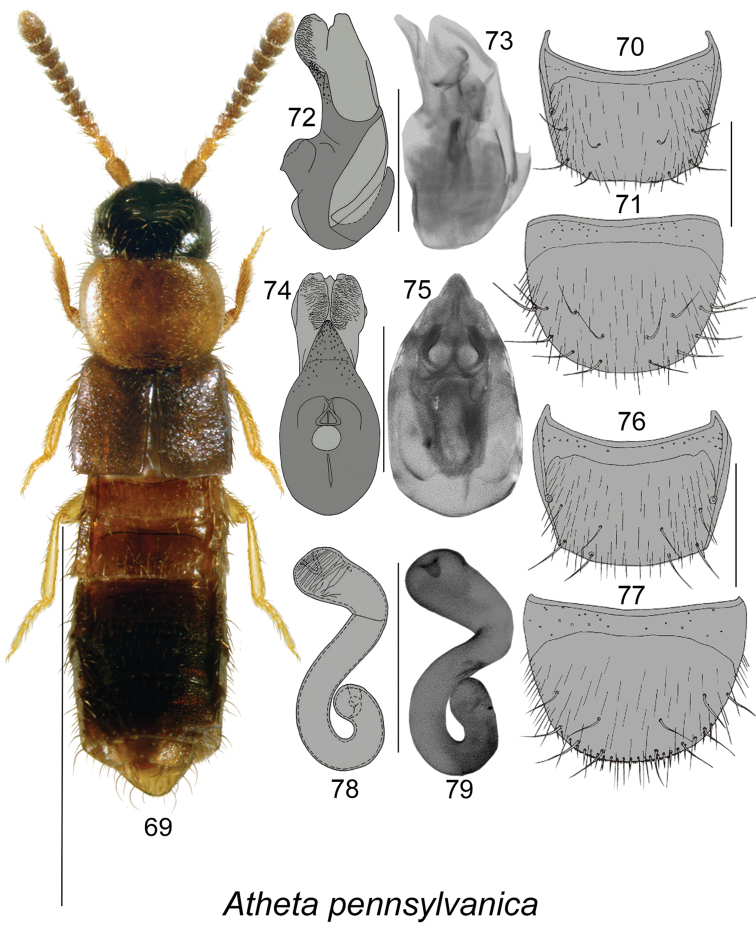
Atheta (Microdota) pennsylvanica Bernhauer: **69** habitus in dorsal view **70** male tergite VIII (after Gusarov 2003b) **71** male sternite VIII (after Gusarov 2003b) **72** median lobe of aedeagus in lateral view (after Gusarov 2003b), and **73** based on Canadian specimen **74** median lobe of aedeagus in ventral view (after Gusarov 2003b), and **75** in dorsal view (based on Canadian specimen) **76** female tergite VIII (after Gusarov 2003b) **77** female sternite VIII (after Gusarov 2003b) **78** spermatheca (after Gusarov 2003b), and **79** based on Canadian specimen. Scale bar for habitus = 1 mm; remaining scale bars = 0.2 mm.

**Figures 80–83.**
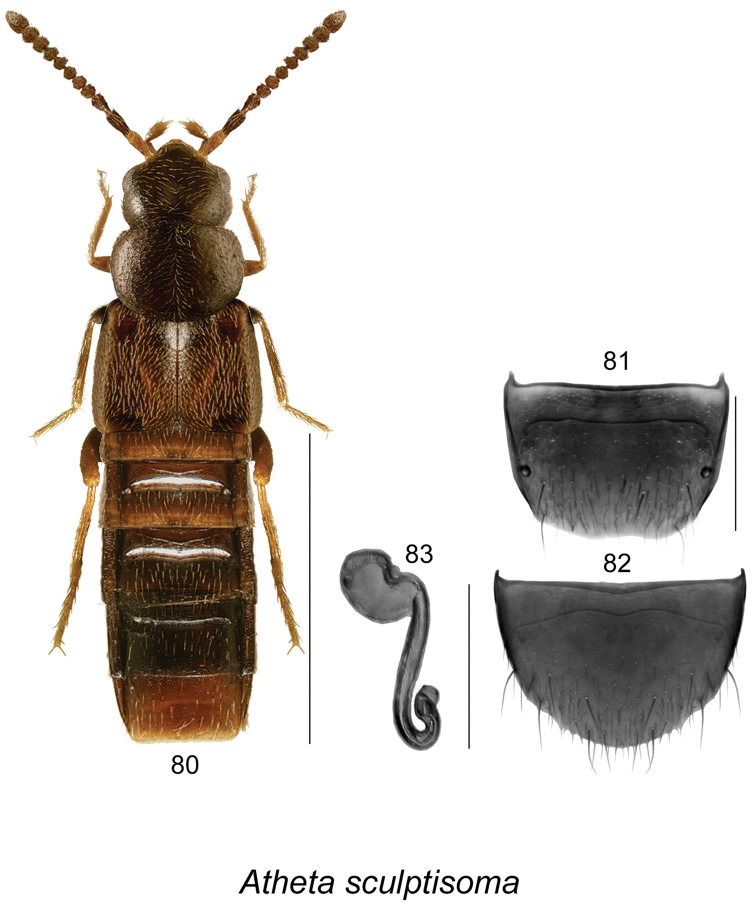
Atheta (Microdota) sculptisoma Klimaszewski & Langor: **80** habitus in dorsal view **81** female tergite VIII **82** female sternite VIII **83** spermatheca. Scale bar for habitus = 1 mm; remaining scale bars = 0.2 mm.

**Figures 84–87.**
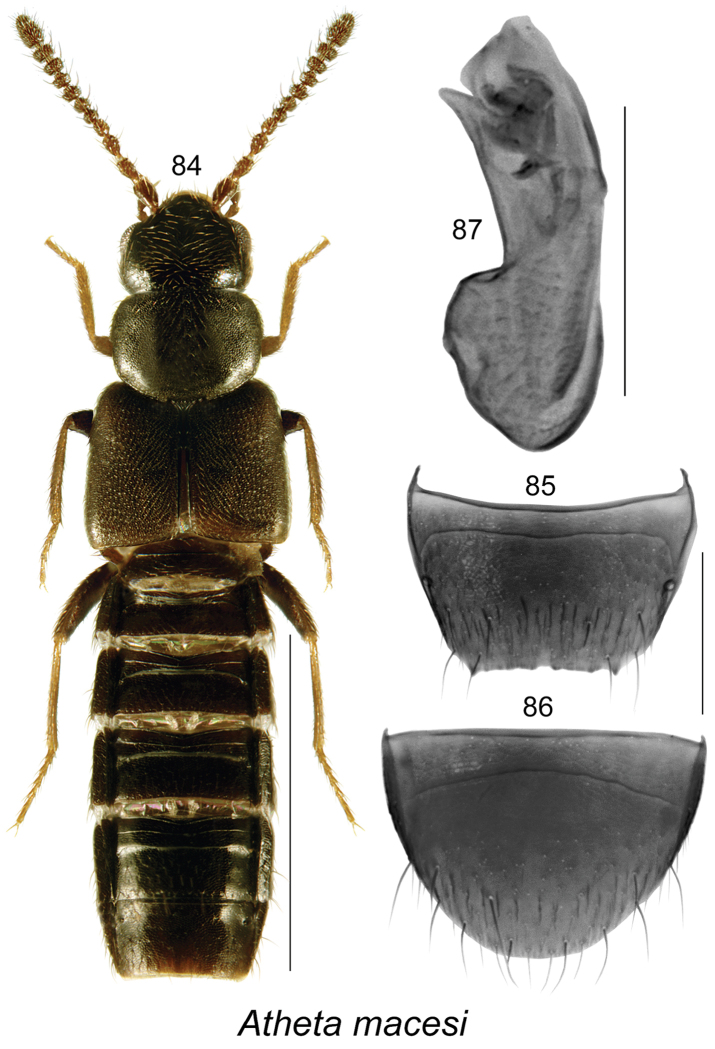
Atheta (Microdota) macesi Klimaszewski & Webster, sp. n.: **84** habitus in dorsal view **85** male tergite VIII **86** male sternite VIII **87** median lobe of aedeagus in lateral view. Scale bar for habitus = 1 mm; remaining scale bars = 0.2 mm.

Some species of *Microdota*, due to small body size and some superficial external similarity, may be confused in collections with members of the subgenus *Datomicra* Mulsant and Rey, from which they may be distinguished by having a fully exposed pronotal hypomeron in lateral view; the pronotal hypomeron is only partially visible in *Datomicra*. Many species of *Datomicra* also have a more densely and coarsely punctate forebody than that of *Microdota*.

*Microdota* may be distinguished from *Dimetrota* by the following combination of characters: body usually parallel-sided, small, on average 2 mm long (*Microdota* – 1.5–2.8 mm; *Dimetrota* – 1.8–3.8 mm, with elytra usually distinctly broader than pronotum); glossae Y-shaped (deeply split in *Dimetrota*); pronotum with sparse to moderately dense and slightly asperate punctation (dense and strongly asperate in *Dimetrota*); lateral margins of pronotum and elytra, and middle and hind tibiae with moderately pronounced macrosetae (strong bristles in *Dimetrota*); hypomera fully visible in lateral view (partially to less often fully visible in *Dimetrota*); and male tergite VIII truncate apically, rarely crenulated, and usually without large lateral teeth (with two large lateral teeth and often distinctive form of margin between them or with pattern of smaller teeth in *Dimetrota*). Details on diagnostics of *Microdota* are provided by Brundin (1948), and Lee and Ahn (2015). Species of Nearctic *Dimetrota* badly need revision.

*Clusiota* may be distinguished from *Microdota* by the following combination of characters: basal antennal article swollen (some species); antennal articles V-X strongly transverse; glossae deeply split medially; pronotum narrower than elytra; elytra flattened, truncate posteriorly and without distinct lateral emargination; abdomen often swollen; spermatheca more or less sinuate with narrowly pear-shaped capsule and small and short apical invagination; and by the median lobe of the aedeagus with large bulbus, strongly ventrally produced tubus bearing elongate subapical part, and with crista apicalis located on elevated part of bulbus.

##### Key to Canadian and Alaskan species of *Microdota*

**Table:** 

1	Elytra at suture about as long as or shorter than pronotum (Figs 9, 17, 25, 56, 61, 69)	**2**
−	Elytra at suture longer than pronotum (Figs 1, 33, 41, 49, 80, 84)	**7**
2	Body bicoloured, head and abdomen dark brown, and pronotum and elytra or elytra only yellowish brown or orange brown (Figs 61, 69); genitalia as illustrated (Figs 64, 65, 68, 72–75, 78, 79)	**3**
−	Body approximately uniformly brown to black; genitalia differently shaped	**4**
3	Pronotum in most specimens orange and elytra yellowish brown (Fig. 69); median lobe of aedeagus and spermatheca as illustrated (Figs 72–75, 78, 79)	**Atheta (Microdota) pennsylvanica Bernhauer**
−	Pronotum brown to light brown and entire elytra or only central part of disc yellowish (Fig. 61); median lobe of aedeagus and spermatheca as illustrated (Figs 64, 65, 68)	**Atheta (Microdota) formicaensis Klimaszewski & Webster, sp. n.**
4	Elytra distinctly broader than maximum width of pronotum (Figs 17, 56); genitalia as illustrated (Figs 20, 21, 24, 59, 60)	**5**
−	Elytra about as wide as maximum width of pronotum (Figs 9, 25); genitalia differently shaped (Figs 12, 13, 16, 28, 29, 32)	**6**
5	Pubescence on forebody sparse, on elytra directed straight posteriad (Fig. 56); spermatheca as illustrated (Figs 59, 60); male unknown	**Atheta (Microdota) festinans (Erichson)**
−	Pubescence on forebody dense, on elytra directed obliquely posteriad (Fig. 17); genitalia as illustrated (Figs 20, 21, 24)	**Atheta (Microdota) curtipenis Klimaszewski & Webster, sp. n.**
6	Pronotum as broad as head (Fig. 25); abdomen subparallel (Fig. 25); antennal articles VI−X moderately transverse (Fig. 25); genitalia as illustrated (Figs 28, 29, 32)	**Atheta (Microdota) microelytrata Klimaszewski & Godin**
−	Pronotum broader than head (Fig. 9); abdomen swollen apically (Fig. 9); antennal articles VI−X strongly transverse (Fig. 9); genitalia as illustrated (Figs 12, 13, 16)	**Atheta (Microdota) pseudosubtilis Klimaszewski & Langor**
7	Elytra almost twice as long as pronotum; median lobe of aedeagus with enlarged oval bulbus and narrow tubus that is ventrally produced at apex; spermatheca S-shaped	**Atheta (Microdota) pratensis (Mäklin)**
−	Elytra 1.2−1.3 times longer than pronotum (Figs 1, 33, 41, 49, 80, 84); genitalia differently shaped	**8**
8	Pronotum dark brown to black and elytra light brown to yellowish-brown (Fig. 49); antennae with articles VII−X strongly transverse, at least twice as wide as long (Fig. 49); pubescence of forebody not soft in appearance; genitalia as illustrated (Figs 51, 52, 55) [males absent in North America]	**Atheta (Microdota) amicula (Stephens)**
−	Pronotum and elytra about the same colour, dark brown with elytra in some specimens only slightly paler (Figs 1, 33, 41, 80, 84) [group of species difficult to distinguish externally]	**9**
9	Elytra 1.2 times broader than pronotum, shoulders moderately angular (Fig. 80); spermatheca with broad, sac-shaped capsule without apparent invagination, stem straight, narrow, half looped posteriorly and slightly twisted at apex (Fig. 83); male unknown	**Atheta (Microdota) sculptisoma Klimaszewski & Langor**
−	Elytra at least 1.3 times broader than pronotum, shoulders strongly angular (Figs 1, 33, 41, 84); spermatheca of a different shape	**10**
10	Pronotal punctation coarse and sparse (Fig. 41); spermatheca with club-shaped capsule bearing deep apical invagination, stem sinuate and twisted apically (Fig. 48); median lobe of aedeagus in lateral view with approximately oval bulbus dorsally (Fig. 45), and sinuate and narrowly elongate tubus laterally (Fig. 44)	**Atheta (Microdota) riparia Klimaszewski & Godin**
−	Pronotal punctation fine and dense (Figs 1, 33, 84); genitalia of a different shape	**11**
11	Body dark brown, almost black, strongly glossy, with dense, meshed and strongly pronounced microsculpture, punctation and pubescence sparse (Fig. 84); male tergite VIII truncate apically, with two small lateral teeth and minute crenulation on apical margin of disc (Fig. 85); median lobe of aedeagus as illustrated (Fig. 87); female unknown	**Atheta (Microdota) macesi Klimaszewski & Webster, sp. n.**
−	Body dark brown, moderately glossy, meshed microsculpture present but not strongly pronounced, punctation and pubescence dense (Figs 1, 33): male tergite VIII and genitalia differently shaped	**12**
12	Pubescence on elytra forming wavy pattern (Fig. 33); median lobe of aedeagus with sinuate venter of tubus in lateral view (Fig. 36), and internal sac with two apical lobes in dorsal view (Fig. 37); spermatheca with tubular capsule, deep and narrow invagination and S-shaped stem (Fig. 40)	**Atheta (Microdota) platonoffi Brundin**
−	Pubescence on elytra directed obliquely posteriad from midline of disc (Fig. 1); median lobe of aedeagus with large bulbus and moderately long tubus, its ventral margin arcuate and narrowly elongate apically (Fig. 4); spermatheca with long, sac-shaped, tubular capsule bearing shallow but broad apical invagination, stem broad and club-shaped (Fig. 8) [adventive in Canada]	**Atheta (Microdota) subtilis (Scriba)**

#### *Subtilis* species group (new)

Species of this group are characterized by: elytra at suture at least as long as pronotum (Figs 1, 9), male tergite VIII truncate and sometimes slightly emarginated medially (Figs 2, 10), median lobe of aedeagus with broadly oval bulbus streamlined with broadly triangular tubus in dorsal view (Figs 5, 13), in lateral view tubus straight medio-basally and narrowly elongate and strongly produced ventrally at apex (Figs 4, 12), internal sac structures complex with two prominent elongate structures in bulbus (Figs 4, 5, 12, 13), spermatheca L-shaped with long, broad sac-shaped capsule bearing wide and shallow apical invagination and with short and swollen apically stem (Figs 8, 16). Two species are known from eastern Canada.

##### Atheta (Microdota) subtilis

Taxon classificationAnimaliaColeopteraStaphylinidae

(Scriba)

Figs 1–8

Homalota subtilis Scriba 1866: 128. As Atheta (Microdota): Brundin 1948, Palm 1970, Benick and Lohse 1974, Smetana 2004 (review of literature and description).

###### Material examined.

**Canada, Labrador**, Goose Bay, Rts. 500 and 520 jct., 53°16.9 N, 60°24.6 W, 13-26.VIII.2001, S. and J. Peck, Flight Intercept Trap, elevation 10 m, spruce-poplar forest (LFC) 3 females, 1 male; Goose Bay, Goose River Bridge, 53°22.2 N, 60°26.2 W, 15-20.VIII.2001, S. and J. Peck, elevation 10 m, spruce-birch forest (LFC) 1 male. **New Brunswick**, **Albert Co.**, Caledonia Gorge P.N.A., 45.7941°N, 64.7736°W, 13.IX.2011, R.P. Webster // near Crooked Creek, mixed forest (red spruce and yellow birch) in decaying gilled mushrooms (RWC) 1 male; Carleton Co., Wakefield, Meduxnekeag Valley Nature Preserve, 46.1940°N, 67.6800°W, 3.VII.2006, R.P. Webster coll. // mixed forest on *Pleurotus* sp. on dead standing *Populus tremuloides* (RWC) 1 male; York Co., New Maryland, Charters Settlement, 45.8331°N, 66.7410°W, 27.VII.2005, R.P. Webster coll. // mixed forest on flowers of *Spiraea alba* (LFC) 1 male; **Restigouche Co.**, off Bellone Road, 47.7755°N, 68.2501°W, 24.VIII.2011, R. Webster and M. Turgeon // Old spruce and fir forest, mossy forest floor, in gilled mushrooms in various stages of decay (RWC) 1 female.

###### Diagnosis.

Body length 1.5−2.0 mm, subparallel, flattened, reddish brown to dark brown, head and abdomen darker than pronotum and elytra in some specimens, legs yellowish brown (Fig. 1); integument moderately glossy, densely punctate and densely pubescent on forebody and less so on abdomen, microsculpture fine; head slightly narrower than pronotum, strongly narrowed posteriad, eyes large and about as long as postocular area dorsally; pronotum transverse, narrower than elytra; elytra wider and longer than pronotum; abdomen subparallel. MALE. Tergite VIII truncate apically (Fig. 2); sternite VIII broadly rounded apically (Fig. 3); median lobe of aedeagus narrow, and strongly ventrally produced apically in lateral view (Fig. 4); internal sac structures complex (Figs 4, 5). FEMALE. Tergite VIII broadly emarginated apically (Fig. 6); sternite VIII slightly emarginated apically (Fig. 7); spermatheca L-shaped with long, broad sac-shaped capsule bearing wide and shallow apical invagination and club-shaped short and swollen apically stem (Fig. 8).

###### Natural history.

The LB specimens were collected in flight intercept traps set in spruce-poplar forest. The NB specimens were found in gilled mushrooms at various stages of decay in spruce/fir forest, in *Pleurotus* sp. on dead standing *Populus tremuloides*, in mixed forest on flowers of *Spiraea alba*, and in a mixed forest with red spruce and yellow birch. Adults were captured from July to September.

###### Distribution.

Atheta (Microdota) subtilis is a Palaearctic species (for details, see Brundin 1948, Palm 1970, Benick and Lohse 1974, Smetana 2004), and it is reported here as adventive for the first time from Canada (LB, NB) and North America.

###### Comments.

Adults of *Atheta subtilis* from LB were captured in association with *Atheta pseudosubtilis* Klimaszewski and Langor. Some females of the former species, because of similarly shaped spermatheca and poorly preserved body, were misidentified as the latter species. We have compared European specimens of *Atheta subtilis* with those from Canada (LB, NB) and found no significant differences in external morphology and shape and structures of genitalia.

##### Atheta (Microdota) pseudosubtilis

Taxon classificationAnimaliaColeopteraStaphylinidae

Klimaszewski & Langor

Figs 9–16

Atheta (Microdota) pseudosubtilis (For diagnosis, see Klimaszewski et al. 2011)

###### Distribution.

Recorded from NF and LB, NB (Klimaszewski et al. 2011, Webster et al. 2012).

###### Comments.

The taxonomic position of *Atheta pseudosubtilis* is somewhat unclear. The shape of the spermatheca is very similar to those of *Atheta subtilis* and *Clusiota antennalis*. Externally it is similar to *Clusiota antennalis* but does not have a swollen basal antennal article. The median lobe of the aedeagus has internal sac structures very similar to those of *Atheta subtilis*. Externally, *Atheta pseudosubtilis* is readily distinguished from *Atheta subtilis* by the much shorter elytra (Figs 1, 9). DNA studies of all these species would be very useful in revealing their true relationships.

#### *Platonoffi* species group (new)

Species of this group are characterized by elytra at suture ranging from shorter to longer than pronotum (Figs 17, 25, 33, 41), male tergite VIII truncate and sometimes slightly emarginated medially or slightly crenulate apically (Figs 18, 26, 34, 42), median lobe of aedeagus with broadly oval bulbus clearly demarcated from triangular tubus in dorsal view (Figs 20, 29, 37, 45), and in lateral view, tubus straight medio-basally, arcuate or sinuate and moderately to strongly produced ventrally at apex (Figs 21, 28, 36, 44), internal sac structures complex (Figs 20, 21, 28, 29, 36, 37, 44, 45); spermatheca S-shaped with long, elongate club-shaped capsule bearing narrow and deep apical invagination and long sinuate stem (Figs 24, 32, 40, 48). Five species are known from Canada and Alaska.

##### Atheta (Microdota) curtipenis

Taxon classificationAnimaliaColeopteraStaphylinidae

Klimaszewski & Webster sp. n.

http://zoobank.org/08F3959E-4933-471F-B4FB-7D29A49665F5

Figs 17–24

###### Holotype (male).

**Canada, New Brunswick, Saint John Co.**, ca 2 km NE of Maces Bay, 45.1168°N, 66.4552°W, 8.V.2006, R.P. Webster, coll. // eastern white cedar swamp, under moose dung (LFC). **Paratypes**: labelled as the holotype (RWC) 1 male, 1 female.

###### Etymology.

The specific name *curtipenis* refers to a short median lobe of aedeagus of this species.

###### Diagnosis.

Body length 2.0–2.3 mm, subparallel, moderately convex, dark brown, abdomen slightly darker than remainder of the body, legs yellowish brown (Fig. 17); integument glossy, densely punctate and densely pubescent on forebody and less so on head and abdomen, microsculpture of forebody fine; head slightly narrower than pronotum, strongly narrowed posteriad and slightly angular posteriorly, eyes large and slightly shorter than postocular area dorsally; pronotum transverse, narrower than elytra; elytra wider and longer than pronotum; abdomen subparallel. MALE. Tergite VIII truncate apically (Fig. 18); sternite VIII broadly rounded apically (Fig. 19); median lobe of aedeagus narrowly oval in dorsal view (Fig. 20), tubus sinuate basally and then straight and rounded apically in lateral view (Fig. 21); internal sac structures as illustrated (Figs 20, 21). FEMALE. Tergite VIII truncate apically (Fig. 22); sternite VIII slightly emarginated apically (Fig. 23); spermatheca compressed S-shaped, capsule spherical with short and narrow apical invagination, stem narrow and U-formed posteriorly (Fig. 24).

###### Natural history.

Adults were found in an eastern white cedar swamp under moose dung in May.

###### Distribution.

Known only from NB, Canada.

###### Comments.

This species is distinguished by the moderately transverse pronotum, and the shape of the median lobe of the aedeagus and spermatheca.

##### Atheta (Microdota) microelytrata

Taxon classificationAnimaliaColeopteraStaphylinidae

Klimaszewski & Godin

Figs 25–32

Atheta (Microdota) microelytrata (For diagnosis, see Klimaszewski et al. 2012)

###### Distribution.

Recorded only from YT (Klimaszewski et al. 2012).

##### Atheta (Microdota) platonoffi

Taxon classificationAnimaliaColeopteraStaphylinidae

Brundin

Figs 33–40

Atheta (Microdota) platonoffi (For diagnosis, see Klimaszewski et al. 2011)

###### Distribution.

In Canada, recorded from AB, BC, LB, NB, NF, NS, ON, SK, YT, and in USA from AK (Klimaszewski et al. 2011, 2015).

##### Atheta (Microdota) pratensis

Taxon classificationAnimaliaColeopteraStaphylinidae

(Mäklin, 1852)

Homalota pratensis Mäklin, 1852: 308. As Atheta (Microdota): Moore and Legner 1975, Klimaszewski et al. 2011; Bousquet et al. 2013.

###### Syntypes.

**USA, Alaska**: Kenai; Holmberg; *pratensis* Mäklin; Mus. Zool. Helsinki, No. 14517 (ZMH) 1 male; same labels except No. 17518 (ZMH) 1 female.

###### Diagnosis.

This species may be readily separated from other Nearctic congeners by the following combination of characters: pronotum rounded and margined, as wide as head and at least 1.5 times narrower than elytra; elytra elongate about twice as long as pronotum with wavy pattern of pubescence posteriorly; male tergite VIII truncate apically; sternite VIII rounded apically; median lobe of aedeagus with large oval bulbus and small triangular tubus in dorsal view, apical part of tubus narrow and produced ventrally in lateral view; female tergite VIII truncate apically, and sternite VIII rounded apically and with antecostal suture sinuate and pointed medially; spermatheca S-shaped with club-shaped capsule bearing deep invagination and sinuate stem looped posteriorly, similar to that of *Atheta platonoffi*.

###### Distribution.

AK, WA (Mäklin 1852, Moore and Legner 1975).

###### Comments.

This species is somewhat similar to *Atheta subtilis* but may readily be distinguished externally by having elytra about 1.5 times wider and almost twice longer than pronotum. We examined the type series but the specimens were in poor shape and therefore were not illustrated.

##### Atheta (Microdota) riparia

Taxon classificationAnimaliaColeopteraStaphylinidae

Klimaszewski & Godin

Figs 41–48

Atheta (Microdota) riparia (For diagnosis, see Klimaszewski et al. 2012)
Atheta (Microdota) riparia Klimaszewski & Godin, 2012: 225.

###### Distribution.

Recorded only from YT in Canada (Klimaszewski et al. 2012).

#### *Pennsylvanica* species group (new)

Species of this group are characterized by elytra at suture ranging from as long as or longer than pronotum (Figs 49, 56, 61, 69), male tergite VIII truncate or slightly emarginated medially and slightly crenulate apically (Figs 50, 70), median lobe of aedeagus with broadly oval bulbus streamlined with broad basally triangular tubus in dorsal view (Figs 52, 65, 74, 75), in lateral view tubus straight and slightly narrowly triangularly produced ventrally (Figs 51, 64, 72, 73), internal sac structures complex (Figs 51, 52, 64, 65, 72–75); spermatheca S-shaped with club-shaped capsule bearing narrow and shallow apical invagination and long, posteriorly looped stem (Figs 55, 59, 60, 68, 78, 79). Four species are known from Canada.

##### Atheta (Microdota) amicula

Taxon classificationAnimaliaColeopteraStaphylinidae

(Stephens)

Figs 49–55

Atheta (Microdota) amicula (For diagnosis, see Klimaszewski et al. 2011, Lee and Ahn 2015)
Aleochara amicula Stephens, 1832: 132. As Atheta (Microdota): Brundin 1948, Palm 1970, Benick and Lohse 1974, Smetana 2004, Lee and Ahn 2015.

###### Distribution.

Atheta (Microdota) amicula is a Palaearctic species adventive in North America. It was reported in Canada based only on female specimens from NF and NS (Majka and Klimaszewski 2008, Klimaszewski et al. 2011). In USA, it was recorded from WA (Moore and Legner 1975). For Palaearctic distribution and synonymy of this species, see Lee and Ahn (2015).

##### Atheta (Microdota) festinans

Taxon classificationAnimaliaColeopteraStaphylinidae

(Erichson)

Figs 56–60

Atheta (Microdota) festinans (For diagnosis and synonymy, see Gusarov 2003b)
Homalota festinans Erichson, 1839: 112. As Atheta (Microdota): Gusarov 2003a, b, Brunke et al. 2012.

###### Material examined.

**Canada, Quebec, Berthier Co.**, Berthierville, 20.XI.2004, Michel Racine coll., sous débris de bois, dans sablière, avec Carabe *Dyschiriodes* sp. (LFC) 1 female.

###### Natural history.

This is the first record with habitat data for this species. The QC specimen was captured from woody debris in a sandy pit in association with *Dyschirius* sp. (Carabidae).

###### Distribution.

Erichson (1839) described this species from PA in USA. Gusarov (2003b) recorded it from AZ, CT, IA, IN, KY, NY, PA, and RI. Bernhauer (1907) reported *Atheta festinans* from ON, and Brunke et al. (2012) confirmed occurrence of this species in Waterloo Reg., ON. Here, we provide the first record of this species from QC.

###### Comments.

All known Canadian specimens of *Atheta festinans* are females. Gusarov (2003b) remarked that all specimens seen of this species were females and suggested that this species may be parthenogenetic.

##### Atheta (Microdota) formicaensis

Taxon classificationAnimaliaColeopteraStaphylinidae

Klimaszewski & Webster sp. n.

http://zoobank.org/A4C2D2A2-735F-4D0B-94B0-D46C3D2BDB8C

Figs 61–68

###### Holotype (male).

**Canada, New Brunswick**, York Co., New Maryland, Charters Settlement, 45.8395°N, 66.7391°W, 19.V.2006, R.P. Webster coll. // mixed forest, on surface of nest of black *Formica* sp. (LFC) 1 male. **Paratypes**: labelled as holotype (RWC) 1 male, 1 female; Charters Settlement, 45.8395°N, 66.7391°W, 29.IV 2004, R.P. Webster coll. // mixed forest, on surface of nest of black *Formica* sp. (RWC) 3 sex undetermined; same data except: 30.IV.2005 // mixed forest in nest of black *Formica* sp., sifting nest material (RWC) 2 sex undetermined; Queens Co., Cranberry Lake P.N.A., 46.1125°N, 65.6075°W, 13.V.2011, R.P. Webster coll. // old red oak forest, in nest of black mound-building *Formica* species, near surface of mound (LFC, RWC) 1 female, 2 sex undetermined.

###### Etymology.

The specific name *formicaensis* is a feminine adjective derived from the generic name *Formica*, an ant genus found in association with the type series.

###### Diagnosis.

Body length 2.6–2.8 mm, subparallel, moderately convex, head and posterior part of abdomen dark brown, pronotum medium to dark brown, elytra with centre of disc yellowish brown and darker edges, base of abdomen light brown, legs yellowish brown (Fig. 61); integument glossy, sparsely punctate and sparsely pubescent, microsculpture distinct and stronger on pronotum and elytra; head slightly narrower than pronotum, rounded and slightly angular posteriorly, eyes small and shorter than postocular area dorsally; antennal articles V–X from subquadrate to slightly transverse; pronotum transverse, slightly narrower than elytra; elytra wider and as long as pronotum; abdomen subparallel. MALE. Tergite VIII truncate apically (Fig. 62); sternite VIII broadly rounded apically and slightly pointed medially (Fig. 63); median lobe of aedeagus narrowly oval in dorsal view with short and triangular tubus (Fig. 65), in lateral view tubus sinuate basally and then straight and rounded apically (Fig. 64); internal sac structures as illustrated (Figs 64, 65). FEMALE. Tergite VIII truncate apically (Fig. 66); sternite VIII broadly rounded apically (Fig. 67); spermatheca small, S-shaped, capsule spherical without apparent apical invagination, stem narrow and sinuate (Fig. 68).

###### Natural history.

Adults were found in association with nests of black ants in the genus *Formica* in April and May.

###### Distribution.

Known only from NB, Canada.

###### Comments.

This species is probably closely associated with nests of the ant genus *Formica*. It is distinguished from all other Nearctic species of *Microdota* by the shape of the median lobe of the aedeagus and spermatheca. The shape of the spermatheca is similar to that of Palaearctic Atheta (Microdota) glabricula Thomson (Palm 1970).

##### Atheta (Microdota) pennsylvanica

Taxon classificationAnimaliaColeopteraStaphylinidae

Bernhauer

Figs 69–79

Atheta (Microdota) pennsylvanica (For diagnosis, see Klimaszewski et al. 2011, and for synonymy, Gusarov 2003b)
Atheta (Microdota) pennsylvanica Bernhauer 1907: 388. As Atheta (Microdota): Gusarov 2003b.

###### Distribution.

This species was recorded in Canada from NB, NF, NS, ON, QC, and in the USA from MN, NY, PA, VT (Gusarov 2003b, Klimaszewski et al. 2011).

#### *Sculptisoma* species group (new)

Species of this group are characterized by elytra at suture at least as long as pronotum (Fig. 80), male unknown; spermatheca pipe-shaped with hemispherical capsule narrowed basally and without apparent apical invagination, and with long stem that is looped posteriorly and twisted apically (Fig. 83). One species belongs to this group.

##### Atheta (Microdota) sculptisoma

Taxon classificationAnimaliaColeopteraStaphylinidae

Klimaszewski & Langor

Figs 80–83

Atheta (Microdota) sculptisoma (For diagnosis, see Klimaszewski et al. 2011)
Atheta (Microdota) sculptisoma Klimaszewski et al. 2011: 148.

###### Distribution.

This native Nearctic species was recorded only from the type locality in southeastern NF in Canada (Klimaszewski et al. 2011).

#### *Macesi* species group (new)

Species of this group are characterized by the strongly glossy body, elytra at suture slightly longer than pronotum (Fig. 84), male tergite VIII truncate apically and with two small lateral teeth (Fig. 85), median lobe of aedeagus with small bulbus and elongate tubus, in lateral view tubus straight, apex narrowly triangular and slightly pointed (Fig. 87), internal sac structures pronounced (Fig. 87); female unknown. One species belongs to this group.

##### Atheta (Microdota) macesi

Taxon classificationAnimaliaColeopteraStaphylinidae

Klimaszewski & Webster sp. n.

http://zoobank.org/A4599D2A-246D-4AD1-A2F4-214C4585DED9

Figs 84–87

###### Holotype (male).

**Canada, New Brunswick, Saint John Co.**, ca 2 km NE of Maces Bay, 45.1161 N, 66.4560 W, 8.V.2006, R.P. Webster, coll. // Eastern white cedar swamp, in sphagnum and litter near brook (LFC).

###### Etymology.

The specific name *macesi* is an adjective derived from Maces Bay in NB, where the holotype specimen was found.

###### Diagnosis.

Body length 2.7 mm, subparallel, flattened, brownish-black, tibiae and tarsi brown (Fig. 84); integument glossy and more so on abdomen, sparsely punctate and pubescent, except for pronotum and elytra; microsculpture of forebody dense and strong, meshed with hexagonal sculpticells; head about as wide as pronotum, slightly angular posteriorly, eyes large and as long as postocular area dorsally; antennae with articles V−X moderately to strongly transverse; pronotum broadest in apical third and narrowest at base, rounded laterally and basally, transverse, narrower than elytra; elytra wider and slightly longer than pronotum; abdomen subparallel. MALE. Tergite VIII truncate apically and with two large lateral teeth (Fig. 85); sternite VIII rounded apically (Fig. 86); median lobe of aedeagus with small bulbus and long tubus, in lateral view tubus straight and apex slightly produced ventrally, apex narrowly triangular and slightly pointed (Fig. 87), internal sac structures well defined (Fig. 87). FEMALE. Unknown.

###### Natural history.

A single male was found in eastern white cedar in sphagnum and litter near a brook, in May.

###### Distribution.

Known only from NB, Canada.

###### Comments.

This species is known only from a single male collected in sphagnum and litter.

### Genus Clusiota

Taxon classificationAnimaliaColeopteraStaphylinidae

Casey

Clusiota Casey, 1910: 119; Moore and Legner 1975: 347.

#### Diagnosis.

**Figures 88–95.**
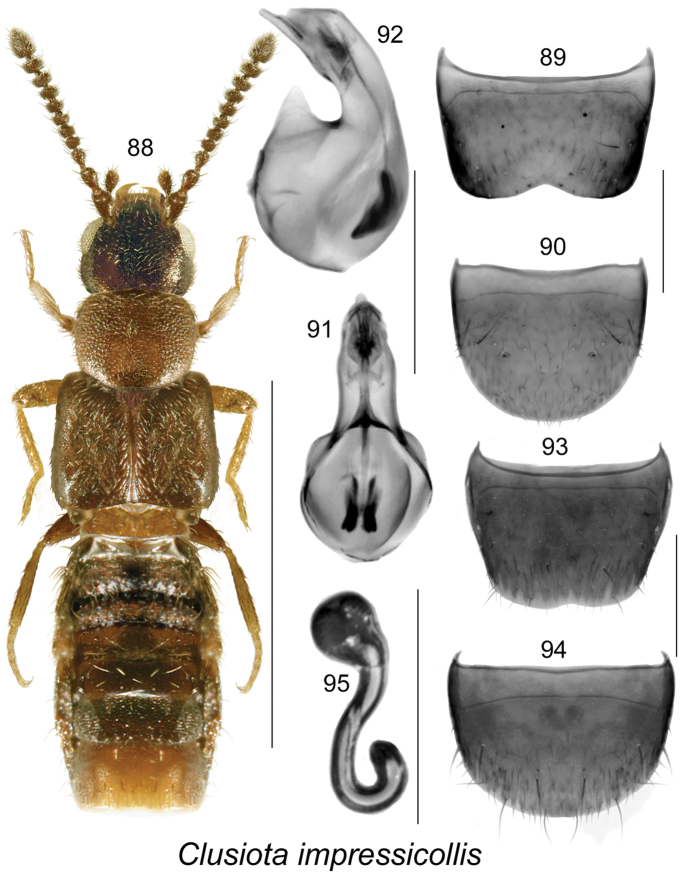
*Clusiota impressicollis* (Bernhauer): **88** habitus in dorsal view **89** male tergite VIII **90** male sternite VIII **91** median lobe of aedeagus in dorsal view **92** median lobe of aedeagus in lateral view **93** female tergite VIII **94** female sternite VIII **95** spermatheca. Scale bar for habitus = 1 mm; remaining scale bars = 0.2 mm.

**Figures 96–103.**
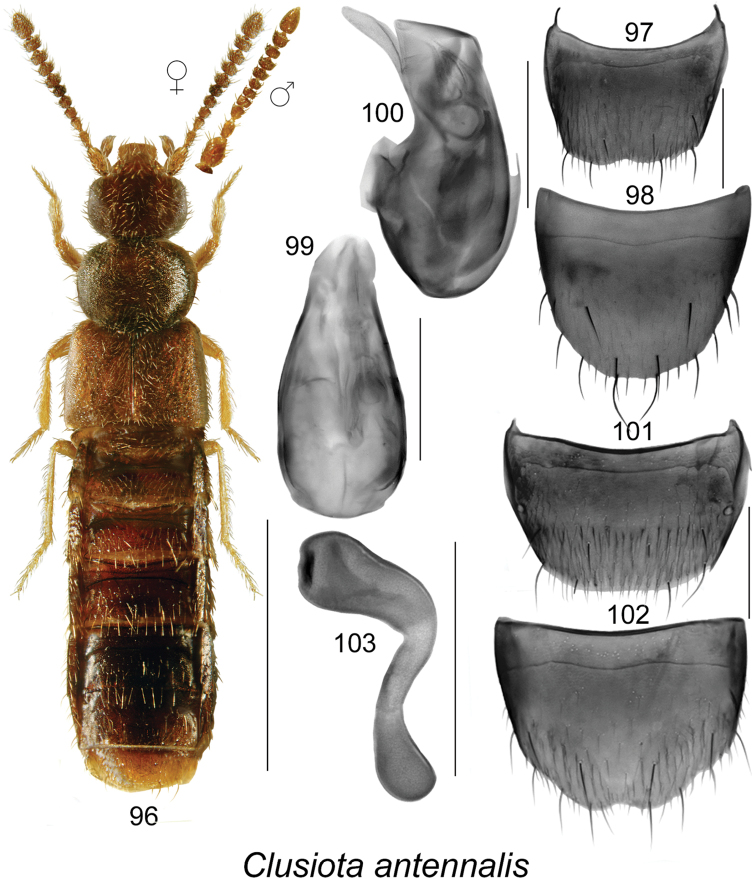
*Clusiota antennalis* (Klimaszewski & Langor): **96** habitus in dorsal view **97** male tergite VIII **98** male sternite VIII **99** median lobe of aedeagus in dorsal view **100** median lobe of aedeagus in lateral view **101** female tergite VIII **102** female sternite VIII **103** spermatheca. Scale bar for habitus = 1 mm; remaining scale bars = 0.2 mm.

**Figures 104–111.**
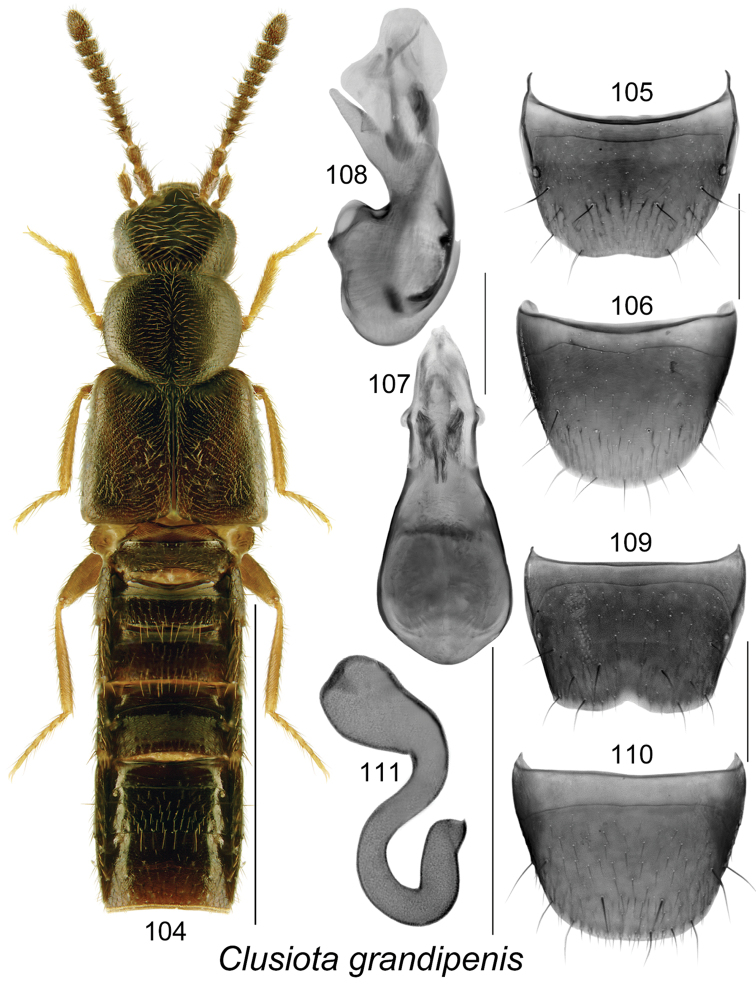
*Clusiota grandipenis* Klimaszewski & Webster, sp. n.: **104** habitus in dorsal view **105** male tergite VIII **106** male sternite VIII **107** median lobe of aedeagus in dorsal view **108** median lobe of aedeagus in lateral view **109** female tergite VIII **110** female sternite VIII **111** spermatheca. Scale bar for habitus = 1 mm; remaining scale bars = 0.2 mm.

The following combination of characters is distinctive for *Clusiota*: small and subparallel body (Figs 96, 88, 104), length 1.5−2.5 mm, antennomere I swollen (Figs 96, 88) except for *Clusiota grandipenis* (Fig. 104), and longer than II, V−X strongly transverse (Figs 96, 88, 104); labial palps with 3 articles; glossae narrow, deeply split forming V-shaped structure; maxillary palpus with 3 articles, last one narrowly elongate; pronotum transverse, about 1.2 times as wide as long, pubescence at midline directed apically in most specimens anteriorly and laterad elsewhere (Figs 96, 88, 104); pronotal hypomeron fully visible medially in lateral view; elytra with pubescence directed obliquely postero-laterad from midline of disc (Figs 96, 88, 104); abdomen slightly swollen posteriorly; male tergite VIII emarginate medially (Figs 89, 97, 105); median lobe of aedeagus with large bulbus and moderately narrow, and triangularly shaped apically tubus in dorsal view (Figs 91, 99, 107), crista apicalis of bulbus large (Figs 92, 100, 108); spermatheca L-shaped or S-shaped with club-shaped tubular capsule, and short sinuate stem (Figs 95, 103, 111).

#### Comments.

Species of this genus may be confused with *Microdota* species, from which they may be readily distinguished by the swollen first basal antennal articles (except for *Clusiota grandipenis*), and shape of genitalia, with median lobe bearing large crista apicalis of bulbus. Casey (1910) believed this genus was related to the subgenus *Datomicra* of *Atheta*.

#### Key to Nearctic species of *Clusiota*

**Table:** 

1.	Elytra longer than pronotum and at least 1.3 times broader than pronotum (Figs 88, 104)	**2**
−	Elytra at most as long as pronotum and about 1.1 times broader than pronotum (Fig. 96); median lobe of aedeagus with tubus straight medially and apical part broad in lateral view (Fig. 100), bulbus without distinct large sclerites in dorsal view (Fig. 99); male tergite VIII with shallowly emarginated apical margin (Fig. 97); spermatheca consisting of club-shaped and elongate capsule and sinuate, short and broad stem (Fig. 103)	***Clusiota antennalis* Klimaszewski & Godin**
2.	Body reddish-brown (Fig. 88); median lobe of aedeagus with tubus sinuate medially and apical part narrow in lateral view (Fig. 92), bulbus with two strong elongate sclerites in dorsal view (Fig. 91); male tergite VIII with deeply emarginated apical margin (Fig. 89); spermatheca sinuate (Fig. 95)	***Clusiota impressicollis* (Bernhauer)**
−	Body dark brown (Fig. 104), median lobe of aedeagus with tubus scarcely sinuate medially and apical part broad in lateral view (Fig. 108), bulbus with differently shaped structures (Fig. 107); spermatheca S-shaped (Fig. 111)	***Clusiota grandipenis* Klimaszewski & Webster, sp. n.**

### Clusiota impressicollis

Taxon classificationAnimaliaColeopteraStaphylinidae

(Bernhauer)

Figs 88–95

Clusiota impressicollis (For diagnosis, see Casey 1910, Klimaszewski et al. 2011)
Atheta impressicollis Bernhauer 1907: 389. As *Clusiota*: Klimaszewski et al. 2011, Brunke et al. 2012, Bousquet et al. 2013.
Clusiota claviventris Casey 1910: 119. Synonymized by Gusarov 2003a.

#### Material examined

**(additional locality data). Canada, Ontario**, Sudbury Co., Mattagami, 25.VIII.1980, R. Baranowski (ZML) 2 males, 1 female; Nipissing Co., Algonquin Provincial Park, near Brent, 21.VIII.1980, R. Baranowski (ZML) 1 male.

#### Distribution.

BC, NB, NF, ON (Casey 1910, Klimaszewski et al. 2011, Bousquet et al. 2013).

#### Natural history.

The specimens from ON were collected in August. The NF specimens were captured in a light flight intercept trap in fir-deciduous forest in July/August (Klimaszewski et al. 2011). These records indicate that adults occur late in the season.

#### Comments.

This species was originally reported by Bernhauer (1907) from Baring, WA and Pasadena, CA. The previously unknown female is illustrated here for the first time from a specimen from Mattagami, ON.

### Clusiota antennalis

Taxon classificationAnimaliaColeopteraStaphylinidae

Klimaszewski & Godin

Figs 96–103

Clusiota antennalis (For diagnosis, see Klimaszewski et al. 2008)
Clusiota antennalis Klimaszewski & Godin in Klimaszewski et al. 2008.

#### Material examined.

**Canada**, **British Columbia**, Cooper River Valley, A31698/F4-1-1, 4.VII–7.VIII.1996, +/- 20 m. pitfall trap, J. Lemieux (LFC) 1 female, 1 sex? same data except: A37541/P2-1-5, 6.VI–4.VII (LFC) 2 females; A36435/04-1-1, 7.VI–6.VII.1996 (LFC) 2 males; Vancouver Island, Mt. Cain: 50.14°N, 126.21°W, 5.VI–27.VI.1996, 16.6 PIT2, N. Winchester (ZML) 2 females; 50.14°N, 126.21°W, 27,VI–13.VII.1966, 16.6.PIT 3, N. Winchester (ZML) 3 females; 50.13°N, 126.21°W, 23.VI–7.VII.1997, 16.6 PIT2, N. Winchester (ZML) 18 females; 50.13°N, 126.21°W, 7.VII–20.VII.1997, 16.6 PIT8, N. Winchester (ZML) 7 females; 50.13°N, 126.21°W, 20.VII–5.VIII.1997, 16.6 PIT 6, N. Winchester (ZML) 1 female; 50.13°N, 126.21°W, +/- 20 m, 1.IX–19.IX.1997, 16.6 PIT8, N. Winchester (ZML) 5 males; same data except: 15.IX–28.IX.1996, 17.4 PAN 3 (ZML) 1 female; 28.IX–12.X.1996, 16.6 PAN 1 (ZML) 3 females; 12.X–1.XI.1996, 16.6 PAN3 (ZML) 1 male; 50.15°N, 126.25°W, 19.IX–4.X.1997, 17.4 PIT 7 (ZML) 1 male, 1 female. **U.S.A.**, **Alaska**, Prince of Wales Is.: Staney Ck., 41–45 m el., 55.79901°N, 133.11782°W, old growth, pitfall 2, 25.VI–9.VII.2012, J. Stockbridge et al. UAM100340147 (UAM) 1 female; same data except: 11–25.VI.2012, UAM100338700 (UAM) 1 female;14–28.V.2012 UAM100338413, UAM100338412, UAM100338414 (UAM) 3 females; Luck Lk. 2 Rd., old growth, 105 m el., 55.96855°N, 132.79615°W, +/- 10 m pitfall 3, 29.VI-8.VII.2010, J. Stockbridge UAM100278064 (UAM) 1 female; Luck Lk. 1 Rd., old growth, 101 m el., 55.97805°N, 132.75456°W, +/- 10 m. pitfall, 8-30.VII.2010, J. Stockbridge, C. Bickford UAM100279634, UAM100279635 (UAM) 2 females; same data except: 27.VI–11.VII.2012 UAM100343219, UAM100343218, UAM100343246 (UAM) 3 females; pitfall 1, 13-27.VI.2012, J. Stockbridge et al. UAM100339932 (UAM) 1 female; Hatchery Ck. 1, 2^nd^ growth, 49 m el., 55.92654°N, 132.95645°N, +/- 10 m pitfall 3, 18.V–4.VI.2010, J. Stockbridge, C. Bickford UAM100262426 (UAM) 1 female; Dall Is., p. 12 el. 688 m., 54.99342°N, 133.01688°N, +/-4 m krummholtz, *Tsuga mertensiana*, *Vaccinium ovalifolium*, pitfall, 15–16.VII.2011, D.S. Sikes UAM100329960, UAM100329969 (UAM) 2 females. UAM data can be downloaded at http://arctos.database.museum/saved/Clusiota_antennalis.

#### Distribution.

Originally described from Dyea, AK (Klimaszewski et al. 2008). It is here newly reported in Canada from BC, and from new localities in AK. *Clusiota antennalis* is a western Nearctic species known only from western BC and AK.

#### Natural history.

In AK, the holotype was captured in alder litter, and the recent AK specimens were collected primarily in pitfall traps from old-growth Pacific rain forests at low elevations although two specimens were collected in an alpine habitat above 650 m elevation. In BC, specimens from Vancouver Island were captured continuously from June to November in pitfall and pan traps.

#### Comments.

Females of this species have a spermatheca extremely similar to those of Atheta (Microdota) subtilis and Atheta (Microdota) pseudosubtilis. *Clusiota antennalis* is easily separated from the two species by the swollen basal antennal article (Fig. 96), and from *Atheta subtilis* by much shorter elytra (Fig. 96). It differs from all Nearctic *Clusiota* and *Microdota* species by the shape of the genitalia.

### Clusiota grandipenis

Taxon classificationAnimaliaColeopteraStaphylinidae

Klimaszewski & Webster sp. n.

http://zoobank.org/0424783F-C865-4166-A976-1D60D3AA10AF

Figs 104–111

#### Holotype (male).

**Canada, New Brunswick**, **Westmorland Co.**, Sackville near Ogden Mill, 45.92155°N, 64.38925°W, 12.V.2006, Scott Makepeace coll. // Black spruce forest, in nest contents of Great Horned Owl – *Bubo virginensis* (LFC). **Paratype**: **Canada, New Brunswick**, **Northumberland Co.**, ca. 2.5 km W of Sevogle, 47.0876N, 65.8613W, 27.VIII.2013, old jack pine forest, in decaying gilled mushroom, R.P. Webster (RWC) 1 female.

#### Etymology.

The specific name *grandipenis*, meaning large penis, refers to the large tubus of the median lobe of the aedeagus of this species.

#### Diagnosis.

Body length 2.2 mm, subparallel, flattened, dark brown, abdomen slightly darker than remainder of the body, legs yellowish brown (Fig. 104); integument glossy, densely punctate and densely pubescent on forebody and less so on head and particularly on abdomen, microsculpture of forebody fine, meshed with hexagonal sculpticells; head about as wide as pronotum, slightly angular posteriorly, eyes large and as long as postocular area dorsally; pronotum rounded laterally and basally, transverse, narrower than elytra; elytra wider and longer than pronotum; abdomen subparallel. MALE. Tergite VIII slightly emarginate apically (Fig. 105); sternite VIII broadly rounded apically (Fig. 106); median lobe of aedeagus with broadly oval bulbus streamlined with apically narrowly triangular tubus in dorsal view (Fig. 107), in lateral view tubus strongly produced ventrally, apex narrowly triangular and slightly pointed (Fig. 108), internal sac structures pronounced (Figs 107, 108). FEMALE. Tergite VIII with shallow apical median emargination (Fig. 109); sternite VIII rounded apically (Fig. 110); spermatheca S-shaped, capsule broadly club-shaped with deep median invagination, stem sinuate with posterior loop (Fig. 111).

#### Natural history.

One adult was found in the nest contents of a Great Horned Owl, – *Bubo virginensis* in a black spruce forest in May and another from a decaying gilled mushroom in a jack pine forest during August.

#### Distribution.

Known only from NB, Canada.

## Supplementary Material

XML Treatment for Microdota

XML Treatment for Atheta (Microdota) subtilis

XML Treatment for Atheta (Microdota) pseudosubtilis

XML Treatment for Atheta (Microdota) curtipenis

XML Treatment for Atheta (Microdota) microelytrata

XML Treatment for Atheta (Microdota) platonoffi

XML Treatment for Atheta (Microdota) pratensis

XML Treatment for Atheta (Microdota) riparia

XML Treatment for Atheta (Microdota) amicula

XML Treatment for Atheta (Microdota) festinans

XML Treatment for Atheta (Microdota) formicaensis

XML Treatment for Atheta (Microdota) pennsylvanica

XML Treatment for Atheta (Microdota) sculptisoma

XML Treatment for Atheta (Microdota) macesi

XML Treatment for Clusiota

XML Treatment for Clusiota impressicollis

XML Treatment for Clusiota antennalis

XML Treatment for Clusiota grandipenis
